# The focus on sample quality: Influence of colon tissue collection on reliability of qPCR data

**DOI:** 10.1038/srep29023

**Published:** 2016-07-07

**Authors:** Vlasta Korenkova, Jana Slyskova, Vendula Novosadova, Sara Pizzamiglio, Lucie Langerova, Jens Bjorkman, Ondrej Vycital, Vaclav Liska, Miroslav Levy, Karel Veskrna, Pavel Vodicka, Ludmila Vodickova, Mikael Kubista, Paolo Verderio

**Affiliations:** 1 Institute of Biotechnology, BIOCEV Centre, Czech Academy of Sciences, Průmyslová 595, 252 42, Vestec u Prahy, Czech Republic; 2 Institute of Experimental Medicine, Czech Academy of Sciences, Prague, Czech Republic; 3 Unit of Medical Statistics, Biometry and Bioinformatics, Fondazione Istituto di Ricovero e Cura a Carattere Scientifico (IRCCS) Istituto Nazionale dei Tumori, Milan, Italy; 4 TATAA Biocenter AB, Göteborg, Sweden; 5 Deparment of Surgery, Teaching Hospital and Medical School Pilsen, Charles University in Prague, Pilsen, Czech Republic; 6 Biomedical Centre, Medical School Pilsen, Charles University in Prague, Pilsen, Czech Republic; 7 Surgical Department, Thomayer Hospital, First Faculty of Medicine, Charles University in Prague, Prague, Czech Republic; 8 Institute of Biology and Medical Genetics, First Faculty of Medicine, Charles University in Prague, Prague, Czech Republic

## Abstract

Successful molecular analyses of human solid tissues require intact biological material with well-preserved nucleic acids, proteins, and other cell structures. Pre-analytical handling, comprising of the collection of material at the operating theatre, is among the first critical steps that influence sample quality. The aim of this study was to compare the experimental outcomes obtained from samples collected and stored by the conventional means of snap freezing and by PAXgene Tissue System (Qiagen). These approaches were evaluated by measuring rRNA and mRNA integrity of the samples (RNA Quality Indicator and Differential Amplification Method) and by gene expression profiling. The collection procedures of the biological material were implemented in two hospitals during colon cancer surgery in order to identify the impact of the collection method on the experimental outcome. Our study shows that the pre-analytical sample handling has a significant effect on the quality of RNA and on the variability of qPCR data. PAXgene collection mode proved to be more easily implemented in the operating room and moreover the quality of RNA obtained from human colon tissues by this method is superior to the one obtained by snap freezing.

Significant effort and funding^1^ are used for the discovery of novel biomarkers and biomarker profiles that play important roles in detecting or predicting specific diseases as well as increasing our understanding of disease mechanisms. Validated biomarkers can reveal a disease from its earliest manifestation and reflect its propagation to the terminal stage in individual patients. This could be most valuable for personalized therapy^2^,^3^. Recent advances in genomics, transcriptomics, proteomics, and other -omics allowed us to generate many more candidate biomarkers than ever before. However, it was shown that published biomarker candidates often show poor reproducibility if tested by different laboratories on patient samples from different clinics^4^,^5^,^6^ or if they are tested in large scales by pharmacological companies. This has triggered efforts for the proper standardization and control of the entire experimental process to minimize the effects of variables that introduce bias and confounding variation^7^.

Experimental measurements based on quantitative analyses, such as gene expression analyses, inevitably require accurate preservation of analysed samples to be able to obtain high quality data. A technical variability in the gene expression measurements can be introduced during different phases of the experimental process. The phases are classified as pre-analytical, analytical, and post-analytical^8^. The pre-analytical phase is defined as “steps starting in chronological order, from the clinician’s request including the examination requisition, a preparation of the patient, a collection of the primary sample, the transportation to and within the laboratory, which ends when the analytical examination procedure begins”, according to ISO 15189:2012. The analytical phase comprises steps of workflow starting in the laboratory and producing measured results. Post-analytical phase is the analysis of obtained results. In recent years, quality assurance tools for improvement of the mainly analytical phase of qPCR experiments have been developed and are described very well in detail in the MIQE guidelines^9^. Because of this, the quality and transparency of the laboratory results have been improved considerably^7^.

Most of the errors are introduced during the pre-analytical phase^10^,^11^. Despite the long-known influence of the pre-analytical phase on the quality of results, it is rarely stringently controlled. One reason is that it occurs outside of the laboratory, usually beyond the control of laboratory personnel. The quality of the sample is already influenced at the operating theatre during the warm and cold ischemia. The warm ischemia is the time between surgical incision and tumour specimen removal^12^, which can trigger the cellular stress response^13^,^14^. Time between tissue removal and its storage, called cold ischemia, should also be minimized because tumour specimen manipulation and storage can both affect the quality of RNA^15^ and can distort the gene expression pattern that is associated with a disease condition within minutes^16^,^17^. For this reason, it is recommended to keep the cold ischemic time short, about 30 minutes maximally^18^. Apart from warm and cold ischemia, there are other sources of possible variability outside the operation theatre, which have been described previously. Among them: the transport of the samples to the laboratory^19^, long-term storage^20^,^21^ or thawing and refreezing of the samples^22^.

Here, we focus on the first part of pre-analytical phase: tissue collection and fixation. A common way to protect the sample is by snap-freezing in liquid nitrogen. Snap-frozen tissue specimens are considered high quality material for molecular analyses and are also preferred for conserving tissue morphology. However, snap-freezing during surgery is complicated since it requires access to liquid nitrogen at the operational theatre. This might not be allowed in some places. An alternative is using a fixative that can be used at room temperature. One option is the PAXgene Tissue System, which is based on usage of the solution that rapidly penetrates and fixes tissue. This technology is compatible with molecular studies in a single sample together with histopathological analyses^23^. The quality of RNA in such preserved tissues is comparable with fresh-frozen tissue and the histology is similar to the one obtained by formalin-fixed paraffin-embedded fixation^24^.

Here, we evaluated the experimental outcomes obtained from samples collected by the conventional snap-freezing and by the new PAXgene Tissue System in two different hospitals.

## Results

### Influence of tissue collection on RNA quality

#### Evaluation of RNA integrity by RNA Quality Indicator (RQI)

The quality of the extracted RNA from all patient samples (see description of samples in the Methods) was determined by RNA Quality Indicator (RQI), which is the method providing integrity measurements of rRNA (28S and 18S region) scaled from 1 to 10^25^ (Supplementary Figures 1–11). It has been described that samples with RNA integrity score higher than 4 reach the quality required for qRT-PCR analysis, while those with RNA integrity score lower than 4 can be applied for amplification of short regions only^20^. Based on this, RQI of 4 was artificially set as a quality borderline value.

**Figure 1:**
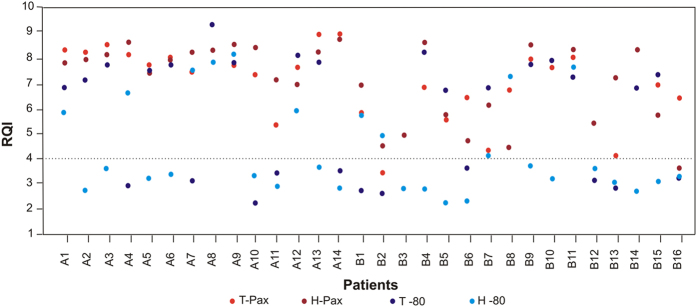
Scatter plot of RQI values measured for each patient, each type of sample and each type of collection method. T-PAX: tumour tissue collected in the PAXgene Tissue System, H-PAX: healthy adjacent tissue collected in the PAXgene Tissue System, T −80: tumour tissue that was frozen, H −80: healthy adjacent tissue that was frozen.

Figure 1 shows a scatter plot of RQI values for each patient, each type of sample (tumour or adjacent healthy tissue) and each type of collection (PAXgene Tissue System fixation or freezing). Seven patients had all 4 samples (tumour PAXgene, tumour freezing, healthy tissue PAXgene, healthy tissue freezing) with a RQI value > 4: 4 from hospital A and 3 from hospital B. All samples (tumour and normal tissue) received from all 14 patients (100%) from hospital A and fixed with PAXgene Tissue System had RQI > 4, while only 4 patients out of 14 (29%) had both paired samples with RQI > 4 when snap-frozen. In hospital B, 12 out of 16 patients (75%) reached RQI > 4 for both tissues when fixed with PAXgene system, and only for 3 patients out of 16 (19%) the quality of frozen paired samples was above the borderline value.

**Figure 2:**
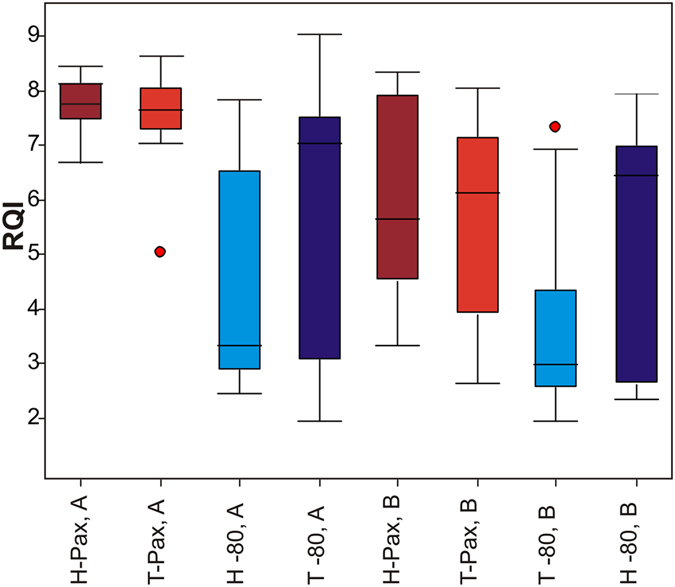
Box plots for the distribution of RQI values divided according to the hospital, the tissue type and the collection method. Box plots represent median and interquartile range. Bars represent the highest and the lowest value excluding outliers, depicted by the dots.

Descriptive statistics of RQI values according to the hospital, the tissue type and the type of collection are listed in Table 1. Box plots (Fig. 2) represent visualizations of distribution among different collection methods for each tissue type in two hospitals. The highest RQI values (means and medians) were obtained from hospital A using PAXgene Tissue System. According to the non-parametric Wilcoxon Signed Rank Test, no significant difference in RQI values was observed between colon cancer tissues and matched adjacent healthy tissues, no matter what preservation mode was used: PAXgene Tissue System in hospital A p-value = 0.47 (n = 14 pairs), PAXgene Tissue System in hospital B p-value = 0.33 (n = 16 pairs), snap-freezing in hospital A p-value = 0.12 (n = 14 pairs), immediate freezing in hospital B p-value = 0.11 (n = 16 pairs). However, in both hospitals the median and mean of RQI values are higher in tumour samples compared to healthy tissue if samples were frozen (Table 1, Fig. 2).

Within each hospital, RQI values of samples which were frozen versus RQI values of samples collected in PAXgene Tissue System were compared by considering both tumor and adjacent healthy tissues (Fig. 2). According to the non-parametric Wilcoxon Signed Rank Test, a statistical significant p-value in both hospitals was obtained (hospital A p-value: < 0.0001 and hospital B p-value: 0.0007), indicating difference in RQI values between collection methods. In hospital A, the median values increased from RQI = 5.85 of frozen samples (IQR = 4.45, n = 28) to RQI = 8.05 of samples fixed in PAXgene Tissue System (IQR = 0.7, n = 28). In hospital B, the median values increased from RQI = 3.6 of frozen samples (IQR = 4.15, n = 32) to RQI = 6.25 of samples fixed in PAXgene Tissue System (IQR = 3.15, n = 32). The yield, purity and quality of each sample using each stabilization method are given in Supplementary Table 1.

#### Evaluation of mRNA integrity using Differential Amplicon Assays

Integrity of mRNA was measured by Differential Amplicon Assay (ΔAMP) approach, which is an independent measure of mRNA quality^26^. The method is based on using paired qPCR assays that produce amplicons of different length (long and short) from the same target. If mRNA is intact, both Cq values should be very similar, if mRNA is degraded then ΔAMP > 0. Acceptable quality of our samples were set to be ΔAMP ≤ 1.0.

**Figure 3:**
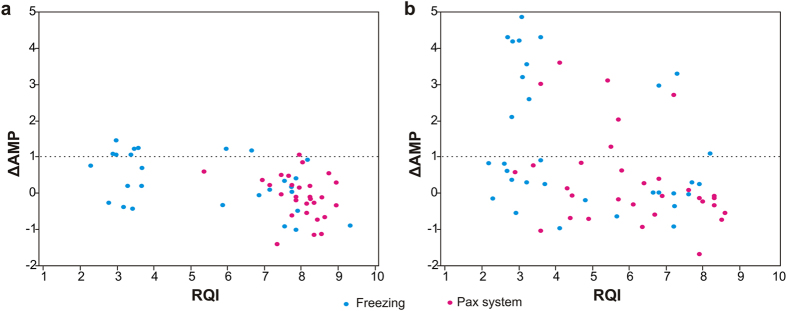
The quality of mRNA determined by ΔAMP assays. Acceptable quality is below ΔCq = 1. (**a**) Samples collected in hospital A. (**b**) Samples collected in hospital B.

Nineteen patients had all 4 samples (tumour PAXgene, tumour freezing, healthy tissue PAXgene, healthy tissue freezing) with ΔAMP ≤ 1.0: 11 from hospital A and 8 from hospital B, irrespectively on tissue type and collection method. All patients (14/14) from hospital A had both paired samples (tumour and adjacent healthy tissue) collected in PAXgene Tissue System with a ΔAMP ≤ 1.0, while 10 patients out of 14 (71%) had both paired samples that were snap-frozen with a ΔAMP ≤ 1.0 (Fig. 3a). Eighty one percent of patients (13/16) from hospital B had both paired samples collected in PAXgene Tissue System with a ΔAMP ≤ 1.0 while 10 patients out of 16 (63%) had both paired samples that were immediately frozen with a ΔAMP ≤ 1.0 (Fig. 3b). Interestingly, several samples with low RQI values (<4) that would be doomed for any regular downstream analysis had ΔAMP ≤ 1.0 (10/28 = 36% collected by snap-freezing, hospital A); 9 samples out of 32 (28%) that were immediately frozen from hospital B and 3 samples out of 32 (9%) collected in PAXgene Tissue System in hospital B. On the contrary, a few samples that would pass quality control with RQI > 4 had ΔAMP > 1.0: 2 samples out of 28 (7%) collected by snap-freezing in hospital A, 2 samples out of 32 (6%) that were immediately frozen in hospital B and 5 samples out of 32 (16%) collected in PAXgene System in hospital B. The scatter plots showing the integrity of RNA of the tissue samples determined by both quality indexes are plotted in Fig. 3a,b. Spearman’s correlation coefficient between the two quality indexes (RQI and ΔAMP) was low, −0.46 (95% CI: −064; −0.22) in Hospital A and −0.43 (95% CI: −0.61; 0.20) in Hospital B. However, using the arbitrary classification according the cut-off values (≥4 for RQI and ≤ 1 for ΔAMP), all samples collected in hospital A using PAXgene Tissue System passed a good quality criteria with both indexes.

In agreement with RQI evaluation, no significant difference in mRNA integrity measured by ΔAMP values was observed between colon cancer tissues and matched adjacent healthy tissues, no matter what preservation mode was used according to the non-parametric Wilcoxon Signed Rank Test: PAXgene Tissue System in hospital A p-value = 0.39 (n = 14 pairs), PAXgene Tissue System in hospital B p-value = 0.25 (n = 16 pairs), snap-freezing in hospital A p-value = 0.80 (n = 14 pairs), immediate freezing in B p-value = 0.32 (n = 16 pairs). The influence of the collection method on RNA quality was significant in both hospitals by considering both tumor and normal samples (Wilcoxon Signed Rank Test p-value = 0.03 in hospital A and p-value = 0.005 in hospital B). The median ΔAMP indicated an improvement in mRNA quality in samples collected in PAXgene Tissue system (median ΔAMP = −0.13 in hospital A and −0.15 in hospital B) with respect to the samples that were frozen (median ΔAMP = 0.14 hospital A and 0.41 in hospital B).

### Influence of tissue collection on stability of gene expression patterns

#### Evaluation of gene expression by single gene analysis

All tested samples of tumours and adjacent healthy tissue from 30 patients (16 from hospital B and 14 from hospital A) were subjected to gene expression profiling using the high-throughput qPCR instrument BioMark (Fluidigm) with 13 already pre-selected assays measuring levels of DNA repair gene expression in the tissue of interest that were normalized with 2 reference genes *TOP1* and *18S* to obtain ΔCq values. The selected transcripts are able to form expression profiles that can distinguish tumour tissue from healthy tissue^27^.

**Figure 4:**
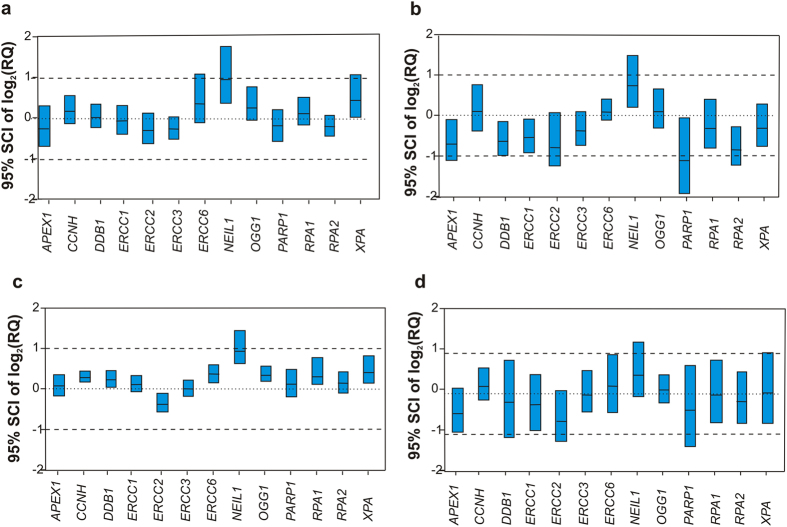
The Simultaneous Confidence Interval (SCI) of the differential expression (ΔΔCq) of each gene in tumour tissue with respect to healthy tissue. (**a**) PAXgene Tissue System, hospital A, (**b**). Snap-freezing, hospital A, (**c**). PAXgene Tissue System, hospital B, (**d**). Immediate freezing, hospital B.

To evaluate impact of tissue collection method on expression profile of 13 individual normalized genes to distinguish tumour tissue from healthy tissue, the computation of the percentile bootstrap simultaneous confidence interval (SCI) for the ΔΔCq value of each gene (ΔCq tumour −ΔCq healthy tissue) was performed^28^. If the intervals contain zero the expression of the specific gene is not different between tumour and normal tissue sample. The results are depicted in Fig. 4a–d.

**Figure 5:**
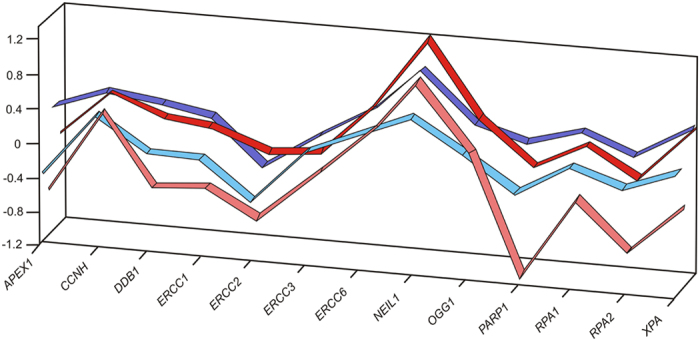
Mean gene expression (ΔΔCq) of differential profiles obtained from both hospitals and both collection methods, respectively, with all tissue samples. Dark red: collection to PAXgene Tissue System in hospital A, light red: snap-freezing in hospital A, dark blue: collection to PAXgene Tissue System in hospital B, light blue: immediate freezing in hospital B.

We observed that in hospital A, there were 2 genes (*NEIL1* and *XPA*) with differential expressions (tumour versus healthy adjacent tissue) collected in PAXgene Tissue System. In the same hospital, significantly different expressions between matched tumour and healthy adjacent tissues were observed for 6 genes (*APEX1*, *DDB1*, *ERCC1*, *NEIL1*, *PARP1*, *RPA2*) after snap freezing collection. In hospital B, gene expression profile differed slightly from expression profiles from hospital A because of the different set of patients. Seven genes out of 13 (*CCNH*, *ERCC2*, *ERCC6*, *NEIL1*, *OGG1*, *RPA1*, *XPA*) had a significantly different expression in tumour versus normal tissue stored in PAXgene Tissue System. Whereas in the matched samples that were immediately frozen, no significantly different expression was measured, probably because of not optimal treatment of the specimens. In addition, by considering the width of the 95% SCI reported in Fig. 4a–d, a higher variability of gene expression for frozen samples (median width of the 95% SCI for hospital A = 0.88 and for hospital B = 1.36) emerges with respect to those collected in PAXgene Tissue System (median width of the 95% SCI for hospital A = 0.49 and for hospital B = 0.37) especially for hospital B. The gene expression pattern is similar for both collection methods in either hospital, respectively (Fig. 5).

#### Evaluation of gene expression using multigene expression patterns

**Figure 6:**
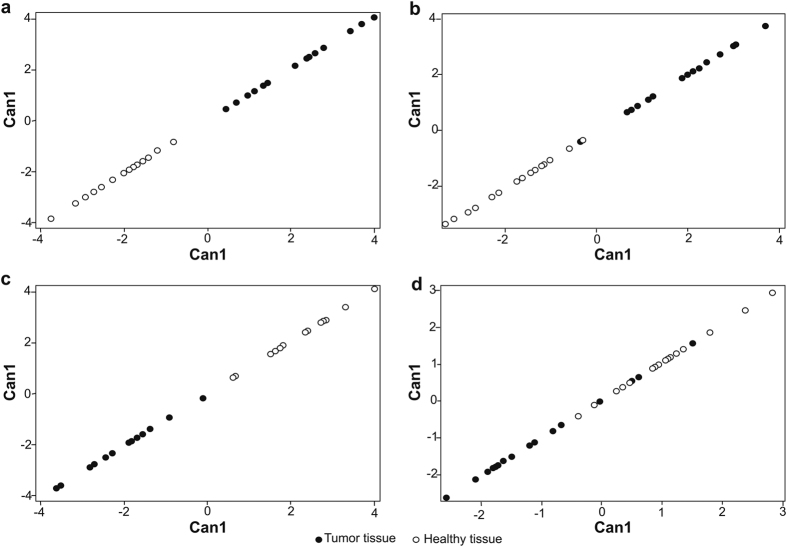
Discriminant analysis of the tumour (black) and the adjacent healthy tissue samples (white). (**a**) Samples collected in hospital A in the PAXgene Tissue System. (**b**) Samples collected in hospital B in the PAXgene Tissue System. (**c**) Samples collected by snap-freezing in hospital A. (**d**) Samples collected by immediate freezing in hospital B. Can1: the first canonical variable – the linear combination of all genes that provides the greatest difference between class means.

All normalized gene expression data (ΔCq) were subjected to discriminant analysis to find out if the gene expression profiles from different hospitals with samples collected under different conditions are able to discriminate tumour tissue from healthy tissue. The analysis showed clear and significant discrimination for samples collected into PAXgene Tissue System in both hospitals A and B (p-value = 0.0021, n = 28 and p-value = 0.0017, n = 32) and for snap-freezing method in hospital A (p-value = 0.0016, n = 28). The immediate freezing in hospital B was not appropriate method to maintain stable gene expression profile that would discriminate tumour tissue from healthy tissue (p-value = 0.19, n = 32) (Fig. 6a–d).

The Squared Mahalanobis Distance (SMD)^29^ was used to assess if removing the samples of lower quality (ΔAMP > 1.0 or RQI < 4) will influence the discrimination ability of tumour tissue samples versus healthy tissue samples . The higher is the value of the SMD, more higher is the discriminatory capability. As worse quality samples were observed mainly with snap-frozen method and with this method specimens were correctly collected only in hospital A, we selected for this evaluation only snap-frozen samples from hospital A. As expected, after removal of ΔAMP > 1.0 or RQI < 4 data, the SMD increased. More specifically, SMD between tumor and healthy tissues with all samples was 18.9 (n = 28), after removing samples with of ΔAMP > 1.0 it increased to 24.2 (n = 24) and when only samples with RQI < 4 were removed the SMD increased to 49.5 (n = 15). It means that the best discrimination was observed when the worse quality samples were excluded according to RQI. On the other hand, if ΔAMP method was performed to identify worse quality samples, less samples had been removed out of multivariate gene expression analysis in order to improve overall discrimination between tumour and heathy tissues.

## Discussion

In this study we evaluated the effect of tissue preservation methods using PAXgene Tissue System and snap-freezing in clinical settings. Our aim was to compare the quality of RNA and gene expression patterns obtained from paired tumour tissue and adjacent healthy colon human tissue from one hospital A and compare our findings with the data from the second hospital B where conditions of collection were not optimal (collection protocols were not exactly followed).

First, the quality of obtained specimens was determined by measurements of RNA integrity using two methods: RNA Quality Index (RQI)^25^ and Differential Amplification (ΔAMP)^26^. RQI or equivalent indexes as RIN (RNA Integrity Number)^30^ reflect the integrity of the dominant ribosomal RNA, which makes up about 85% of the total RNA amount. rRNAs are chemically and structurally different from mRNA and thus differently respond to different degrading agents and consequently to different treatments^31^. Moreover, degraded samples show larger variation and substantial uncertainty below integrity number 5^26^.

In order to complete the whole picture, the integrity of mRNA using ΔAMP was measured^26^. The principle of ΔAMP is based on the evaluation of the ratio of paired amplicons of different length amplified from the same target. If mRNA is intact, Cqs of both assays are the same, while for degraded RNA, Cq of the longer amplicon is higher due to the lower yield^31^. This method should reflect changes caused by mRNA degradation more sensitively than RQI evaluation. All samples collected by hospital staff into PAXgene Tissue System in the hospital A, exactly according to the protocol, displayed a good integrity of RNA, it means ΔAMP ≤ 1.0, RQI > 4, and the highest RQI values: median RQI = 8.2 in healthy tissue samples and median RQI = 7.9 in tumour tissue samples. In the hospital B, where the PAXgene collection protocol was partially modified, about a quarter of paired samples did not pass quality criteria and the mean RQI values were lower than in hospital A: median RQI = 5.9 in healthy tissue samples and mean RQI = 6.4 in tumour tissue samples. The RQI of our samples collected in PAXgene Tissue System are comparable to the values published in literature for a snap-frozen tissue. For example, mean RQI for snap-frozen human colon tissues was about 7.7 or mean RIN (RNA integrity number) about 7.2^32^. In another example, the mean RIN value of 7.5 was recorded in human colon samples that were snap frozen within 10 minutes after extraction, 30 minutes after extraction mean RIN was 6.7, and 90 minutes after extraction mean RIN dropped to 4.2^15^. If we compared our PAXgene results with published integrity numbers for snap-frozen tissues, then we would comply with the conclusions of the comparative study of Viertler *at al*.^23^, who determined that PAXgene-fixed rat liver and kidney tissues provided RNA quantity and quality similar to that from snap-frozen tissue in the laboratory conditions. However, the quality of our RNA isolated from the human colon tissues that were immediately frozen after extraction, was significantly worse than the quality of our matched samples collected in PAXgene Tissue System. Tissue samples collected by snap-freezing in hospital A, exactly according to the protocol, had median RQI = 3.6 in healthy tissue samples and median RQI = 7.3 in tumour tissue samples. Quality criteria were not fulfilled for almost 3/4 of paired samples (at least one sample from the pair was under the quality criteria). The RNA with the worst quality was isolated from the frozen samples from hospital B. The deviation of protocol was the most substantial. Samples were not snap-frozen, instead they were immediately inserted in −80 °C freezer. This kind of immediate freezing is actually a slow freezing process. During this process, the core of the larger sample freeze later compared to outer surfaces, which may lead to variation in RNA quality in different parts of the sample^19^,^33^. Healthy tissue samples, collected by this mean in the hospital B, had mean RQI = 3.3 and tumour tissue samples exhibited mean RQI = 6.8. More than 3/4 quarters of paired samples did not pass RQI or ΔAMP quality criteria.

We further observed that there was no significant difference between integrity (RQI and ΔAMP) of RNA isolated from tumour tissues and adjacent healthy tissues using either collection method. Specifically, when samples were snap-frozen or immediately frozen, RNA integrity of adjacent healthy tissues was lower than in tumour tissues, which is in partial agreement with previous finding of Bao^20^, who described these differences as significant because of the different composition of tumour and healthy tissue. If PAXgene Tissue System was applied, the median integrity values for both tissues were almost identical. This indicates the rapid and efficient biomolecule preservation with the PAXgene fixative solution. The similar observation was made previously using another type of fixative RNA Later RNA Stabilization Reagent during collection of resected colorectal tissues: no significant differences in mean RIN scores between the normal and tumour samples were observed^17^.

The link between the lower quality of the samples and their higher gene expression variability has already been established^25^,^34^. It has also been evidenced that RNA quality has a noticeable influence on the significance of differential expression of individual marker genes between two divergent risk groups of cancer patients^35^, which could be summed in the well-known sentence: Rubbish in, rubbish out. Our results comply with these conclusions. We have observed that even though mean differential gene expression patterns obtained for matched samples by 2 different collection means within the same hospital are similar, significance of differential expression of individual genes differs as well as quality of RNA. Only *NEIL1* gene was able to significantly distinguish tumour tissue from healthy tissue by both collection methods. Significance of differential expression of individual marker genes as well as variability could be influenced by the collection mode, quality of RNA, different sets of patients in two hospitals and relatively small number of patients. Thus, univariate analysis of expression changes between tumour tissue and healthy tissue with small number of patients and small fold changes of differential gene expression (less than 2) will not provide us with definitive outcomes and it should be combined with results of multivariate analysis. Nevertheless, what we can observe from our univariate analysis is the width of simultaneous confidence intervals obtained for each collection method that can be linked to various quality of RNA.

The widest SCIs were obtained for tissue samples collected by immediate freezing in the hospital B where there was no difference in expression of any analyzed gene between normal and tumour tissue observed. A higher variability in individual gene expression values could cause wiping out any significant differences between tumour and healthy tissues. The lowest median RNA integrity values were measured in these specimens, 63% of all samples did not pass quality criteria RQI or ΔAMP. Also, multivariate discriminatory analysis using 13 gene classifier, was not able to discriminate tumour tissue samples from adjacent healthy samples. On the basis of our results and previous publications^19^,^33^, we do not recommend this kind of freezing for samples that are aimed for gene expression analysis.

On the other hand, tissue specimens that were collected by conventional snap-freezing into the liquid nitrogen and then replaced to −80 °C freezer, were suitable to discriminate the tumour and healthy samples with multivariate discriminate analysis in spite of the lower quality of RNA (54% of all samples did not pass the quality criteria RQI or ΔAMP). The widths of the SCI of individual genes were comparable to SCI of specimens collected into PAXgene Tissue System in the same hospital A. The snap-frozen samples were also used to asses whether the exclusion of samples with worse RNA quality improved the discriminatory ability of the multipanel gene expression. SMD showed that discrimination of healthy tissue samples from tumour tissue samples by gene expression profiling improved after exclusion of 14% samples (ΔAMP > 1.0). Improvement was higher if 46% of samples were removed according to RQI < 4. Even though exclusion of the precious patient samples from analysis is a painful step for researcher to do, it is known that the quality of biological samples ultimately determines the quality of any analysis performed with these samples^19^,^35^. The exclusion of lower quality RNA samples is necessary for accurate diagnosis, prediction of outcome, for selection of appropriate therapy or the molecular characterization of human diseases.

All presented evidence proves that PAXgene-fixed colon tissues provided RNA quality significantly better than that obtained from snap-frozen tissues collected in clinical setting in both hospitals. Using multigene classifier, it was possible to significantly discriminate tumour tissue from adjacent healthy tissue when fixed with PAXgene Tissue System. Low variability of gene expression was observed, thus this approach enables us to reliably detect smaller fold changes of gene expression. PAXgene collection mode proves to be a good option for the operating theatres where use of liquid nitrogen is restricted.

## Methods

### Human tissue samples

Two hospitals in the Czech Republic participated in the study, collecting tissue specimens from patients having surgery of colon carcinoma. Two samples were collected from each patients; tumour tissue and adjacent healthy colon tissue (5–10 cm distant from the tumour). Collection of human samples was approved by ethical committees of participated hospitals and the methods were carried out in accordance with the approved guidelines (Ethics committee at the Institute of Clinical and Experimental medicine and Thomayer Hospital, approved on April 13th 2011, and Ethics committee at the Teaching Hospital and Medical School in Pilsen, approved on July 11th 2012) and all study participants have signed informed consent. In total 120 patient samples were collected and included in the study. Paired tumour and adjacent healthy tissues were collected by different means from 14 and 16 patients from hospital A and B, respectively. The same tissue specimen (tumour or adjacent healthy tissue) was divided in two pieces, one was collected in PAXgene Tissue System (Qiagen), and the second piece was fresh-frozen and further stored at −80 °C. The protocol using PAXgene Tissue System was as follows: tissue was placed into the PAXgene Tissue Container and preserved in the PAXgene Tissue FIX solution for up to 24 hours at room temperature, which was then replaced by the PAXgene Tissue Stabilizer Concentrate and stored at −80 °C. All specimens were kept at −80 °C until isolation. Warm ischemic time during surgery varied between 5–20 minutes, while the cold ischemic time took no longer than 5 minutes in all cases. The maximal size of the tissue was recommended to be ≤2 cm^3^.

Despite the fact that the detailed protocol was distributed to the hospitals, some deviations in collection and processing of the tissue samples between the two hospitals were discovered after collection: 1. rinsing of the specimen to get rid of stool with warm tap water in hospital B only. 2. in hospital B, liquid nitrogen was not available at the place of surgery, instead, the sample was inserted in a cryo tube and within 5 minutes stored in a −80 °C freezer present in the same room. In hospital A, the sample was inserted in a cryo tube and snap-frozen in liquid nitrogen within 5 minutes. 3. in hospital B, the specimen was inserted into the PAXgene Tissue FIX container according to the manufacturer’s protocol. Within 5 minutes, the sample was stored at −80 °C in the fixative instead of removing the PAXgene Tissue FIX after 24 hours and replacing it by PAXgene Tissue Stabilizer. Despite the protocol deviation, we decided to investigate the quality of these specimens because PAXgene Tissue FIX solution is designed to quickly fix the tissue and stabilize the RNA, thus gene expression profile could be retained.

### Isolation of RNA

Tissue samples were homogenized in the MagNA Lyser (Hoffmann-La Roche). AllPrep DNA/RNA mini kit (Qiagen) was used to isolate nucleic acids from the samples. RNA from tissues collected in PAXgene containers was extracted using the PAXgene Tissue RNA Kit (PreAnalytiX) according to the manufacturer’s instructions.

### Quantity and quality control of RNA

RNA quantity and purity was measured with Nanodrop spectrophotometer ND-1000 (Thermo Scientific) and RNA integrity was measured with Experion Automated Electrophoresis System (Bio-Rad) with Experion RNA StdSence Analysis Kit (Bio-Rad). Information on the samples is included in Supplementary Table S1.

### qPCR assay design and validation

Primer/probe assays with PerfectProbe were purchased from Primer Design. Specificity and efficiency of all assays were tested. Information on all primers and their validation is included in Supplementary Table S2. The transcripts are functionally divided: transcripts from base excision repair pathway: *OGG1*, *APEX1*, *NEIL1*, *PARP1*, transcripts from nucleotide excision pathway: *XPA*, *RPA1*, *RPA2*, *ERCC3* (*XPB*), *ERCC2* (*XPD*), *ERCC1*, *ERCC6* (*CSB*), *DDB1*, *CCNH*. For normalization, 2 reference genes were used (*TOP1*, *18 S*), which had been tested previously using the same type of tissue material^27^ and evaluated with Normfinder (GenEx, MultiD Analyses).

### Reverse transcription

cDNA was synthesized from 50 ng of RNA in 10 μl reaction using a RevertAidTM First strand cDNA synthesis kit (MBI Fermentas) using random hexamers and following manufacturer’s instructions. cDNA samples were stored at −20 °C and diluted just before use 1:1 with RNase-free water.

### Testing integrity of mRNA by Differential Amplicons (ΔAMP) Method

Possible degradation of mRNA was evaluated by applying a new method for evaluation of integrity. The ΔAMP method^26^ uses three assay sets (Assay set 1–3) for integrity analysis of RNA. Each set has 3 assay variants with various amplicon size (74–342 bp) named short (S), medium (M) and long (L) assays. Assays in each set have one primer in common within the set (forward or reverse). The length of ΔAMP assays was selected to be of similar length as assays used for expression profiling. Long (L) and Short (S) assays of assay set 3 were selected for calculating ΔAMP value for each sample: ΔAMP = Cq_L_ − Cq_S_. The quality cut off value was set to +1.0. The 10 μl qPCR reaction contained 5 μl of TATAA SYBR GrandMaster Mix (TATAA Biocenter), 2 μl of cDNA, 0.2 μl of mixed reverse and forward primers with a final concentration of 200 nM and 2.8 μl of water. Temperature profile was 95 °C for 30 s for polymerase activation and 40 cycles of 95 °C for 10 s, 58 °C for 10 s and 72 °C for 35 s. Melting curve analysis followed. The qPCR reactions were run in CFX384 qPCR cycler (Bio-Rad).

### High-throughput qPCR

Each sample was pre-amplified 18 cycles with a mix of 15 primer pairs (without *18 S*). The reaction contained 10 μl of iQ Supermix (Bio-Rad), 4 μl of cDNA, 2 μl of pooled primers with a final concentration of each primer of 25 nM and 4 μl of water. Temperature profile was 95 °C for 15 s and 60 °C for 4 min. As a control, NTC was included in the pre-amplification reaction, one extra sample was included as IPC. The pre-amplified cDNA was immediately used or placed in freezer at −20 °C. The pre-amplified cDNA was diluted 10x with water prior to the use. qPCR was performed using the high-throughput platform BioMark™ HD System (Fluidigm) and two 48.48 GE Dynamic Arrays. Five μL of sample pre-mix contained 1 μL of 10× diluted pre-amplified cDNA, 2.5 μL of Taqman universal mastermix II without UNG (Applied Biosystems), 0.25 μL of 20× GE sample loading reagent (Fluidigm) and 1.25 μL of water. Five μL of assay pre-mix contained 1.25 μL of 12 μM primer/probe assays with PerfectProbeTM (Primer Design) with final concentration of 300 nM in reaction, 2.5 μL of 2× assay loading reagent (Fluidigm). Thermal conditions for qPCR were: 95 °C for 10 min, 35 cycles of 95 °C for 15 s and 60 °C for 60 s.

### Data pre-processing

Gene expression data were collected from two GE Dynamic Arrays 48 × 48. IPC was used to recalculate the background fluorescence from two arrays at the same level. Cq cut off was set up to 25 and values higher than 25 were replaced by the value of 25 (Cq = 25 in BioMark correspond approximately to Cq = 35 in a conventional qPCR cycler)^36^. Data were normalized to reference genes (*18 S* and *TOP1*) to obtain ΔCq values: ΔCq = (Cq gene − mean Cq of the two reference genes). All data were pre-processed in GenEx Enterprise (MultiD Analyses).

### Statistical analysis

Statistical analyses were performed using SAS software v. 9.2. (SAS Institute Inc.), GeneEx Enterprise (MultiD Analyses) and SigmaPlot 13.0.

#### Comparison of sample integrity in PAXgene Tissue System versus freezing

Difference between RQI or ΔAMP values obtained in colon cancer tissue and matched normal tissue within each hospital were evaluated by resorting to non-parametric approach (Wilcoxon Signed Rank Test). The same approach was used to assess the difference between RQI or ΔAMP values obtained in tissues collected in PAXgene Tissue System versus those collected by snap-freezing. The correlation between the two integrity indexes was assessed by the Spearman Correlation Coefficient and its 95% confidence interval (CI) obtained using the Fisher’s transformation.

#### Effect of tissue collection method on stability of gene expression patterns

Simultaneous Confidence Interval (SCI): For each considered gene the relevance of the expression changes between tumour tissue and adjacent healthy tissue were evaluated by computing the 95% SCI for the ΔΔCq value of each gene (ΔCq tumour −ΔCq healthy tissue) within each collection method and hospital. If the intervals contain zero the expression of the specific gene is not significantly different between tumour and normal tissue sample. This approach^37^ takes into consideration the simultaneous determination of all the markers on the same set of subjects.

Linear Discriminant Analysis (LDA): In order to jointly consider the expression change of all the gene between tumour and normal tissue within each collection method and hospital, LDA was resorted. This technique provides a linear combination (i.e. canonical correlation) of the gene expression that maximize the separation between normal and tumour tissue^38^ by assuming a multivariate normal distribution within each group, with a common covariance matrix. Inference was made by testing the null hypothesis that the first canonical correlation is equal to zero.

The Squared Mahalanobis Distance (SMD)^29^ was used in order to describe how removing of samples with quality indexes ΔAMP > 1.0 or RQI < 4 influences the discrimination of tumour tissue samples from healthy adjacent tissue samples. The SMD was computed by using a pooled covariance matrix.

## Additional Information

**How to cite this article**: Korenkova, V. *et al*. The focus on sample quality: Influence of colon tissue collection on reliability of qPCR data. *Sci. Rep.* **6**, 29023; doi: 10.1038/srep29023 (2016).

## Supplementary Material

Supplementary Information

## Figures and Tables

**Table 1:** Descriptive statistics for RQI values according to the hospital, the tissue type and the mean of collection.

Hospital	Collection mode	Status of tissue	Num. of samples	Mean RQI	STD	maximum	median	minimum	IQR
A	PAXgene Tissue System	healthy	14	8.0	0.6	8.7	8.2	6.9	0.6
		tumour	14	7.8	0.9	8.9	7.9	5.3	0.7
	Snap-freezing	healthy	14	4.8	2.0	8.1	3.6	2.7	3.4
		tumour	14	6.1	2.4	9.3	7.3	2.2	4.4
B	PAXgene Tissue System	healthy	16	6.3	1.7	8.6	5.9	3.6	3.3
		tumour	16	6.0	1.8	8.3	6.4	2.9	3.1
	Freezing	healthy	16	3.9	1.6	7.6	3.3	2.2	1.6
		tumour	16	5.4	2.3	8.2	6.8	2.6	4.3

STD: standard deviation, IQR: interquartile range.

## References

[b1] Markets and Markets. Cancer/Tumor Profiling Market worth $35.03 Billion by 2018. Available at: http://www.marketsandmarkets.com/PressReleases/cancer-tumor-profiling.asp (Accessed: 19^th^ January 2016) (2016).

[b2] Moorcraft S. Y., Smyth E. C. & Cunningham D. The role of personalized medicine in metastatic colorectal cancer: an evolving landscape. Therapeutic advances in gastroenterology 6, 381–395 (2013). 24003339 10.1177/1756283X13491797 PMC3756633

[b3] Mohelnikova-Duchonova B., Melichar B. & Soucek P. FOLFOX/FOLFIRI pharmacogenetics: The call for a personalized approach in colorectal cancer therapy. World journal of gastroenterology 20, 10316–10330 (2014). 25132748 10.3748/wjg.v20.i30.10316 PMC4130839

[b4] Dougherty E. R. Biomarker development: prudence, risk, and reproducibility. BioEssays: news and reviews in molecular, cellular and developmental biology 34, 277–279 (2012). 10.1002/bies.201200003 22337590

[b5] McShane L. M. & Polley M. Y. Development of omics-based clinical tests for prognosis and therapy selection: the challenge of achieving statistical robustness and clinical utility. Clinical trials 10, 653–665 (2013). 24000377 10.1177/1740774513499458 PMC4410005

[b6] Hayes D. F. . Breaking a vicious cycle. Science translational medicine 5, 196CM6 (2013). 10.1126/scitranslmed.3005950 23903752

[b7] Johnson G., Nour A. A., Nolan T., Huggett J. & Bustin S. Minimum information necessary for quantitative real-time PCR experiments. Methods in molecular biology 1160, 5–17, (2014). 24740217 10.1007/978-1-4939-0733-5_2

[b8] Verderio P. Assessing the clinical relevance of oncogenic pathways in neoadjuvant breast cancer. Journal of clinical oncology 30, 1912–1915 (2012). 22508829 10.1200/JCO.2012.41.7386

[b9] Bustin S. A. . The MIQE guidelines: minimum information for publication of quantitative real-time PCR experiments. Clinical chemistry 55, 611–622 (2009). 19246619 10.1373/clinchem.2008.112797

[b10] Carraro P. & Plebani M. Errors in a stat laboratory: types and frequencies 10 years later. Clinical chemistry 53, 1338–1342 (2007). 17525103 10.1373/clinchem.2007.088344

[b11] Kristensen G. B., Aakre K. M., Kristoffersen A. H. & Sandberg S. How to conduct External Quality Assessment Schemes for the pre-analytical phase? Biochemia medica 24, 114–122 (2014). 24627720 10.11613/BM.2014.013 PMC3936964

[b12] Ma Y., Dai H. & Kong X. Impact of warm ischemia on gene expression analysis in surgically removed biosamples. Analytical biochemistry 423, 229–235 (2012). 22343191 10.1016/j.ab.2012.02.003

[b13] Lin D. W. . Influence of surgical manipulation on prostate gene expression: implications for molecular correlates of treatment effects and disease prognosis. Journal of clinical oncology 24, 3763–3770 (2006). 16822846 10.1200/JCO.2005.05.1458

[b14] Musella V. . Effects of warm ischemic time on gene expression profiling in colorectal cancer tissues and normal mucosa. PloS one 8, e53406 (2013). 23308215 10.1371/journal.pone.0053406 PMC3538764

[b15] Hong S. H. . Effects of delay in the snap freezing of colorectal cancer tissues on the quality of DNA and RNA. Journal of the Korean Society of Coloproctology 26, 316–323 (2010). 21152133 10.3393/jksc.2010.26.5.316 PMC2998017

[b16] Spruessel A. . Tissue ischemia time affects gene and protein expression patterns within minutes following surgical tumor excision. BioTechniques 36, 1030–1037 (2004). 15211754 10.2144/04366RR04

[b17] Yamagishi A. . Gene profiling and bioinformatics analyses reveal time course differential gene expression in surgically resected colorectal tissues. Oncology reports 31, 1531–1538 (2014). 24573535 10.3892/or.2014.3053 PMC3975991

[b18] Viana C. R. . The interference of cold ischemia time in the quality of total RNA from frozen tumor samples. Cell and tissue banking 14, 167–173 (2013). 22562477 10.1007/s10561-012-9313-5

[b19] Kap M. . The influence of tissue procurement procedures on RNA integrity, gene expression, and morphology in porcine and human liver tissue. Biopreservation and biobanking 13, 200–206 (2015). 26035010 10.1089/bio.2014.0076

[b20] Bao W. G. . Biobanking of fresh-frozen human colon tissues: impact of tissue *ex-vivo* ischemia times and storage periods on RNA quality. Annals of surgical oncology 20, 1737–1744 (2013). 22711177 10.1245/s10434-012-2440-1

[b21] Kap M., Oomen M., Arshad S., de Jong B. & Riegman P. Fit for purpose frozen tissue collections by RNA integrity number-based quality control assurance at the Erasmus MC tissue bank. Biopreservation and biobanking 12, 81–90 (2014). 24749874 10.1089/bio.2013.0051

[b22] Ma Y., Dai H., Kong X. & Wang L. Impact of thawing on reference gene expression stability in renal cell carcinoma samples. Diagnostic molecular pathology 21, 157–163 (2012). 22847160 10.1097/PDM.0b013e31824d3435

[b23] Viertler C. . A new technology for stabilization of biomolecules in tissues for combined histological and molecular analyses. The Journal of molecular diagnostics 14, 458–466 (2012). 22749745 10.1016/j.jmoldx.2012.05.002

[b24] Groelz D. . Non-formalin fixative versus formalin-fixed tissue: a comparison of histology and RNA quality. Experimental and molecular pathology 94, 188–194 (2013). 22814231 10.1016/j.yexmp.2012.07.002

[b25] Fleige S. & Pfaffl M. W. RNA integrity and the effect on the real-time qRT-PCR performance. Molecular aspects of medicine 27, 126–139 (2006). 16469371 10.1016/j.mam.2005.12.003

[b26] Björkman J., Švec D., Lott E., Kubista M. & Sjöback R. Differential amplicons (ΔAmp)—a new molecular method to assess RNA integrity. Biomolecular Detection and Quantification 6, 4–12 (2016). 27077042 10.1016/j.bdq.2015.09.002 PMC4822209

[b27] Slyskova J. . Functional, Genetic, and Epigenetic Aspects of Base and Nucleotide Excision Repair in Colorectal Carcinomas. Clin Cancer Res. 18, 5878–5887 (2012). 22966016 10.1158/1078-0432.CCR-12-1380

[b28] Mandel M. & Betensky R. A. Simultaneous Confidence Intervals Based on the Percentile Bootstrap Approach. Computational statistics & data analysis 52, 2158–2165 (2008). 19137059 10.1016/j.csda.2007.07.005 PMC2440720

[b29] McLachlan G. J. Mahalanobis distance. Resonance 4, 20–26 (1999).

[b30] Schroeder A. . The RIN: an RNA integrity number for assigning integrity values to RNA measurements. BMC molecular biology 7, 3 (2006). 16448564 10.1186/1471-2199-7-3 PMC1413964

[b31] Kubista M., Bjorkman J., Svec D. & Sjoback R. RNA quality matters. *European Pharmaceutical Review* 17, 63–67 (2012).

[b32] Denisov V., Strong W., Gingrich J. & Wintz H. Development and Validation of RQI: An RNA Quality Indicator for the Experion™ Automated Electrophoresis System. Electrophoresis tech note 5761. Available at: http://www.gene-quantification.com/Bio-Rad-bulletin-5761.pdf (Accessed: 19^th^ January 2016) (2008).

[b33] Scouten C. W. Frozen Section Technique in the Animal Research Setting in *A Practical Guide to Frozen Section Technique* (ed Peters S. R.) 171–191 (Springer, 2010).

[b34] Die J. V. & Roman B. RNA quality assessment: a view from plant qPCR studies. Journal of experimental botany 63, 6069–6077 (2012). 23045609 10.1093/jxb/ers276

[b35] Vermeulen J. . Measurable impact of RNA quality on gene expression results from quantitative PCR. Nucleic acids research 39, e63 (2011). 21317187 10.1093/nar/gkr065 PMC3089491

[b36] Korenkova V. . Pre-amplification in the context of high-throughput qPCR gene expression experiment. BMC molecular biology 16, 5 (2015). 25888347 10.1186/s12867-015-0033-9 PMC4365555

[b37] Pizzamiglio S. . Simultaneous confidence intervals to compare gene expression profiles using ABC transporter TaqMan microfluidic cards. Oncology reports 23, 853–860 (2010). 20127029

[b38] Gutierrez-Osuna R. L10: Linear discriminants analysis. Available at: http://research.cs.tamu.edu/prism/lectures/pr/pr_l10.pdf (Accessed: 19^th^ January 2016) (2016).

