# Supplementary material for: The focus on sample quality: Influence of colon tissue collection on reliability of qPCR data
Source: Sci Rep. 2016 Jul 7;6:29023. doi: 10.1038/srep29023 (PMC4935944; doi:10.1038/srep29023)
Supplement: Supplementary Information [file srep29023-s1.doc]

**The focus on sample quality: Influence of colon tissue collection on reliability of qPCR data**

Vlasta Korenkova^1^*, Jana Slyskova^2^, Vendula Novosadova^1^, Sara Pizzamiglio^3^, Lucie Langerova^1^, Jens Bjorkman^4^, Ondrej Vycital^5,6^, Vaclav Liska^5,6^, Miroslav Levy^7^, Karel Veskrna^7^, Pavel Vodicka^2,6,8^, Ludmila Vodickova^2,6,8^, Mikael Kubista^1,4^, Paolo Verderio^3^

*^1^Institute of Biotechnology, BIOCEV Centre, Czech Academy of Sciences, Průmyslová 595, 252 42, Vestec u Prahy, Czech Republic,* [*Vlasta.korenkova@ibt.cas.cz*](mailto:Vlasta.korenkova@ibt.cas.cz)*, +420 721 732 643*

*^2^Institute of Experimental Medicine, Czech Academy of Sciences, Prague, Czech Republic*

*^3^Unit of Medical Statistics, Biometry and Bioinformatics, Fondazione Istituto di Ricovero e Cura a Carattere Scientifico (IRCCS) Istituto Nazionale dei Tumori, Milan, Italy*

*^4^TATAA Biocenter AB, Göteborg, Sweden*

*^5^Deparment of Surgery, Teaching Hospital and Medical School Pilsen, Charles University in Prague, Pilsen, Czech Republic*

*^6^Biomedical Centre, Medical School Pilsen, Charles University in Prague, Pilsen, Czech Republic*

*^7^Surgical Department, Thomayer Hospital, First Faculty of Medicine, Charles University in Prague, Prague, Czech Republic*

*^8^Institute of Biology and Medical Genetics, First Faculty of Medicine, Charles University in Prague, Prague, Czech Republic*

**Supplementary Table 1.** List of the samples with description of their quality, quantity and integrity.

| **ΔAMP ≥ 1.0** |
| --- |
| **RQI < 4.0** |

| **ID** | **260/280** | **260/230** | **ng/ul** | **RQI** | **ΔAMP** | **ID** | **260/280** | **260/230** | **ng/ul** | **RQI** | **ΔAMP** |
| --- | --- | --- | --- | --- | --- | --- | --- | --- | --- | --- | --- |
| **Hospital A** |  |  |  |  |  |  |  |  |  |  |  |
| **A1 H-80** | 2.1 | 1.2 | 486.9 | **5.8** | **-0.4** | **A1 H-Pax** | 2.0 | 1.7 | 192.7 | **7.8** | **-0.2** |
| **A1 T-80** | 2.1 | 1.0 | 247.8 | **6.8** | **-0.1** | **A1 T-Pax** | 2.0 | 1.6 | 1259.4 | **8.3** | **-0.3** |
| **A2 H-80** | 2.1 | 2.1 | 751.8 | **2.7** | **-0.3** | **A2 H-Pax** | 2.0 | 1.4 | 227.4 | **7.9** | **0.1** |
| **A2 T-80** | 2.0 | 2.1 | 3356.0 | **7.1** | **0.1** | **A2 T-Pax** | 2.0 | 1.6 | 178.2 | **8.2** | **-0.2** |
| **A3 H-80** | 2.1 | 2.2 | 239.7 | **3.6** | **0.1** | **A3 H-Pax** | 2.1 | 1.7 | 295.4 | **8.1** | **-0.6** |
| **A3 T-80** | 2.1 | 1.8 | 408.7 | **7.7** | **0.0** | **A3 T-Pax** | 2.0 | 1.8 | 326.3 | **8.5** | **-0.1** |
| **A4 H-80** | 2.1 | 1.6 | 264.6 | **6.6** | **1.1** | **A4 H-Pax** | 2.1 | 1.9 | 252.6 | **8.6** | **-0.7** |
| **A4 T-80** | 2.1 | 1.8 | 456.4 | **2.9** | **1.0** | **A4 T-Pax** | 2.1 | 1.9 | 1072.4 | **8.1** | **-0.3** |
| **A5 H-80** | 2.1 | 2.0 | 395.0 | **3.2** | **0.1** | **A5 H-Pax** | 1.7 | 0.7 | 18.2 | **7.4** | **-0.1** |
| **A5 T-80** | 2.1 | 2.0 | 487.5 | **7.5** | **0.3** | **A5 T-Pax** | 2.0 | 1.5 | 79.5 | **7.7** | **0.2** |
| **A6 H-80** | 2.1 | 1.3 | 382.5 | **3.4** | **-0.5** | **A6 H-Pax** | 2.1 | 1.9 | 287.1 | **7.9** | **1.0** |
| **A6 T-80** | 2.2 | 1.5 | 511.5 | **7.7** | **0.1** | **A6 T-Pax** | 2.0 | 1.7 | 378.6 | **8.0** | **0.8** |
| **A7 H-80** | 2.1 | 1.5 | 377.2 | **7.5** | **-1.0** | **A7 H-Pax** | 2.2 | 2.0 | 262.7 | **8.2** | **-0.2** |
| **A7 T-80** | 1.8 | 0.1 | 4.9 | **3.1** | **-0.4** | **A7 T-Pax** | 2.2 | 2.4 | 92.0 | **7.4** | **0.5** |
| **A8 H-80** | 2.1 | 1.4 | 178.8 | **7.8** | **-1.0** | **A8 H-Pax** | 2.1 | 2.1 | 420.7 | **8.3** | **-1.2** |
| **A8 T-80** | 2.1 | 1.3 | 156.1 | **9.3** | **-0.9** | **A8 T-Pax** | 2.1 | 1.9 | 746.3 | **7.8** | **-0.2** |
| **A9 H-80** | 2.1 | 1.3 | 116.8 | **8.1** | **-0.3** | **A9 H-Pax** | 2.1 | 1.8 | 234.2 | **8.5** | **-1.2** |
| **A9 T-80** | 2.1 | 0.8 | 230.4 | **7.8** | **-0.5** | **A9 T-Pax** | 2.1 | 2.0 | 362.2 | **7.7** | **-0.7** |
| **A10 H-80** | 1.9 | 0.4 | 30.6 | **3.3** | **1.0** | **A10 H-Pax** | 2.1 | 1.8 | 329.0 | **8.4** | **-0.8** |
| **A10 T-80** | 1.9 | 0.1 | 23.6 | **2.2** | **0.7** | **A10 T-Pax** | 2.2 | 1.1 | 130.9 | **7.3** | **-1.4** |
| **A11 H-80** | 2.0 | 1.3 | 82.7 | **2.9** | **1.4** | **A11 H-Pax** | 1.9 | 1.1 | 67.9 | **7.1** | **0.2** |
| **A11 T-80** | 2.0 | 1.1 | 267.9 | **3.4** | **1.1** | **A11 T-Pax** | 1.8 | 0.8 | 26.8 | **5.3** | **0.5** |
| **A12 H-80** | 2.0 | 1.7 | 370.1 | **5.9** | **1.2** | **A12 H-Pax** | 1.9 | 0.5 | 39.3 | **6.9** | **0.3** |
| **A12 T-80** | 2.1 | 0.8 | 565.8 | **8.1** | **0.8** | **A12 T-Pax** | 1.9 | 1.5 | 52.5 | **7.6** | **0.4** |
| **A13 H-80** | 1.8 | 0.9 | 169.8 | **3.6** | **0.6** | **A13 H-Pax** | 2.0 | 0.6 | 75.7 | **8.2** | **0.1** |
| **A13 T-80** | 2.0 | 1.9 | 805.5 | **7.8** | **0.3** | **A13 T-Pax** | 2.0 | 2.0 | 149.1 | **8.9** | **-0.4** |
| **A14 H-80** | 2.1 | 1.9 | 181.5 | **2.8** | **1.0** | **A14 H-Pax** | 2.1 | 0.6 | 48.3 | **8.7** | **0.5** |
| **A14 T-80** | 2.1 | 1.0 | 275.1 | **3.5** | **1.2** | **A14 T-Pax** | 2.1 | 1.8 | 260.7 | **8.9** | **0.2** |
| **ID** | **260/280** | **260/230** | **ng/ul** | **RQI** | **ΔAMP** | **ID** | **260/280** | **260/230** | **ng/ul** | **RQI** | **ΔAMP** |
| **Hospital B** |  |  |  |  |  |  |  |  |  |  |  |
| **B1 H-80** | 2.0 | 1.1 | 122.0 | **5.7** | **-0.7** | **B1 H-Pax** | 2.0 | 1.6 | 266.8 | **6.9** | **-0.1** |
| **B1 T-80** | 2.0 | 0.4 | 51.8 | **2.7** | **0.5** | **B1 T-Pax** | 2.1 | 1.8 | 206.9 | **5.8** | **0.5** |
| **B2 H-80** | 2.0 | 0.4 | 29.1 | **4.8** | **-0.3** | **B2 H-Pax** | 2.0 | 1.9 | 168.0 | **4.5** | **-0.2** |
| **B2 T-80** | 2.0 | 2.2 | 586.8 | **2.6** | **0.7** | **B2 T-Pax** | 2.1 | 1.7 | 247.6 | **3.4** | **0.7** |
| **B3 H-80** | 2.0 | 1.2 | 382.7 | **2.9** | **-0.6** | **B3 H-Pax** | 2.0 | 1.9 | 141.9 | **4.9** | **-0.8** |
| **B3 T-80** | 2.0 | 1.8 | 253.3 | **2.8** | **0.3** | **B3 T-Pax** | 2.0 | 0.2 | 14.0 | **2.9** | **0.5** |
| **B4 H-80** | 2.0 | 2.1 | 642.2 | **2.8** | **2.0** | **B4 H-Pax** | 2.0 | 1.4 | 63.2 | **8.6** | **-0.6** |
| **B4 T-80** | 2.0 | 1.3 | 632.3 | **8.2** | **1.0** | **B4 T-Pax** | 2.0 | 1.8 | 127.4 | **6.8** | **0.3** |
| **B5 H-80** | 2.0 | 2.1 | 538.5 | **2.2** | **0.7** | **B5 H-Pax** | 2.1 | 1.6 | 350.6 | **5.7** | **1.9** |
| **B5 T-80** | 2.1 | 2.2 | 2463.2 | **6.7** | **-0.1** | **B5 T-Pax** | 2.0 | 2.0 | 429.1 | **5.5** | **1.2** |
| **B6 H-80** | 2.1 | 0.9 | 340.9 | **2.3** | **-0.2** | **B6 H-Pax** | 2.1 | 2.0 | 227.9 | **4.7** | **0.7** |
| **B6 T-80** | 2.1 | 2.2 | 1553.9 | **3.6** | **0.8** | **B6 T-Pax** | 2.1 | 1.7 | 868.5 | **6.4** | **0.2** |
| **B7 H-80** | 2.0 | 0.2 | 62.9 | **4.1** | **-1.0** | **B7 H-Pax** | 2.1 | 1.9 | 210.2 | **6.1** | **-0.4** |
| **B7 T-80** | 2.1 | 1.4 | 339.8 | **6.8** | **-0.1** | **B7 T-Pax** | 2.1 | 1.9 | 293.9 | **4.3** | **0.1** |
| **B8 H-80** | 2.1 | 1.4 | 530.1 | **7.2** | **-0.5** | **B8 H-Pax** | 2.1 | 1.4 | 165.9 | **4.4** | **-0.8** |
| **B8 T-80** | 2.1 | 1.5 | 1065.8 | **7.2** | **-1.0** | **B8 T-Pax** | 2.1 | 1.8 | 767.2 | **6.7** | **-0.7** |
| **B9 H-80** | 2.1 | 1.4 | 531.1 | **3.7** | **0.2** | **B9 H-Pax** | 2.2 | 1.8 | 153.2 | **8.5** | **-0.8** |
| **B9 T-80** | 2.0 | 1.9 | 1568.7 | **7.7** | **0.2** | **B9 T-Pax** | 2.1 | 1.5 | 370.7 | **7.9** | **-1.7** |
| **B10 H-80** | 2.1 | 1.4 | 340.4 | **3.2** | **0.2** | **B10 H-Pax** | 2.2 | 1.4 | 117.1 | **7.9** | **-0.2** |
| **B10 T-80** | 2.1 | 1.3 | 764.9 | **7.9** | **0.2** | **B10 T-Pax** | 2.1 | 2.1 | 287.9 | **7.6** | **0.0** |
| **B11 H-80** | 2.1 | 0.8 | 480.7 | **7.6** | **-0.1** | **B11 H-Pax** | 2.2 | 1.8 | 204.2 | **8.3** | **-0.2** |
| **B11 T-80** | 2.1 | 2.0 | 1211.7 | **7.2** | **-0.1** | **B11 T-Pax** | 2.1 | 2.0 | 1016.6 | **8.0** | **-0.3** |
| **B12 H-80** | 2.0 | 0.4 | 64.0 | **3.6** | **4.1** | **B12 H-Pax** | 1.7 | 1.1 | 10.2 | **5.4** | **2.9** |
| **B12 T-80** | 2.1 | 1.6 | 167.9 | **3.1** | **4.6** | **B12 T-Pax** | 2.2 | 1.4 | 16.5 | **3.6** | **2.9** |
| **B13 H-80** | 2.1 | 1.5 | 327.0 | **3.0** | **4.0** | **B13 H-Pax** | 2.0 | 1.4 | 54.9 | **7.2** | **2.5** |
| **B13 T-80** | 2.0 | 0.4 | 34.2 | **2.8** | **4.0** | **B13 T-Pax** | 2.0 | 1.3 | 14.9 | **4.1** | **3.4** |
| **B14 H-80** | 1.9 | 0.3 | 75.0 | **2.7** | **4.1** | **B14 H-Pax** | 2.0 | 1.2 | 33.2 | **8.3** | **-0.1** |
| **B14 T-80** | 2.0 | 1.4 | 1016.0 | **6.8** | **2.8** | **B14 T-Pax** | 2.0 | 1.7 | 46.9 | **8.3** | **-0.4** |
| **B15 H-80** | 2.0 | 1.2 | 395.5 | **3.1** | **3.1** | **B15 H-Pax** | 2.0 | 2.1 | 11.0 | **5.7** | **-0.2** |
| **B15 T-80** | 2.1 | 1.4 | 596.4 | **7.3** | **3.1** | **B15 T-Pax** | 2.1 | 1.1 | 125.3 | **6.9** | **-0.2** |
| **B16 H-80** | 2.0 | 1.8 | 239.0 | **3.3** | **2.4** | **B16 H-Pax** | 2.0 | 0.3 | 12.2 | **3.6** | **-1.1** |
| **B16 T-80** | 2.1 | 0.3 | 57.4 | **3.2** | **3.4** | **B16 T-Pax** | 1.8 | 0.3 | 7.7 | **6.4** | **-1.0** |

**Supplementary Table 2.** Description of the primers.

| **Gene symbol** | **Gene description** | **refseq** | **amplicon lenght** | **Forward primer sequence** | **Reverse primer sequence** | **Efficiency (%)** | **r2** | **NTC (Cq)** | **Repeatibility**  **(SD)** |
| --- | --- | --- | --- | --- | --- | --- | --- | --- | --- |
| *APEX1* | base excision repair pathway, AP edonuclease | NM 001641 | 115 | TGCCTTCAAGAGACCAAATGTT | CGCCACTGTACCCTTCCTT | 0.97 | 0.993 | x | 0,04 |
| *CCNH* | nucleotide excision pathway, kinase subunits of TFIIH CCNH | NM 001239 | 107 | GGGGTACGGGTGTTTTACG | GCTTCTGACTACTGTTGTGGTA | 0.90 | 0.996 | x | 0,02 |
| *DDB1* | nucleotide excision pathway, complex defective in XP goup E DDB1 | NM 001923 | 150 | TGCTGGGAGACATGGAAGG | CAACACCATTATCAAGGTATGTCAAG | 0.90 | 0.994 | x | 0,07 |
| *ERCC1* | nucleotide excision pathway, 5’ incision DNA binding subunit | NM 001983 | 124 | CAATCCCGTACTGAAGTTCGT | TGGGTGCAGGTTGTGGTAG | 1.02 | 0.997 | x | 0,08 |
| *ERCC2 (XPD)* | nucleotide excision pathway, 5’ to 3’ DNA helicase | NM 000400 | 130 | CCAAAGGCTTCACCATCATCA | ACAGACTGGAAACGCTCAAATA |  |  | x | 0,06 |
| *ERCC3 (XPB)* | nucleotide excision pathway, 3’ to 5’ DNA helicase | NM 000122 | 122 | GAAAGTTGCCACCCTGATGTA | GATTTGCTTGTGAAAGTCTCTGT | 0.92 | 0.991 | x | 0,1 |
| *ERCC6 (CSB)* | nucleotide excision pathway, cockayne syndrome and UV-Sensitive Syndrome; needed for transcription-coupled NER ERCC6 | NM 000124 | 129 | ATGCGTGGATTGTCGTCTTC | TTCTTGTTCTTCCTCTGCTTCTT | 1.04 | 0.968 | x | 0,12 |
| *NEIL1* | base excision repair pathway, removes thymine glycol | NM 024608 | 95 | GACTGGCGCTTTCTGATTTC | AGCCAAGCAACAACAACAAC | 1.00 | 0.958 | x | 0,33 |
| *OGG1* | base excision repair pathway, 8-oxoG opposite C | NM 016821 | 101 | TGGGGCATCGTACTCTAGC | AGATTGTCCAGAAGGCAGAAC | 1.07 | 0.995 | x | 0,07 |
| *PARP1* | base excision repair pathway, protects strand interruptions | NM 001618 | 113 | CCACACACAATGCGTATGACT | CCACAGCAATCTTCGGTTATGA | 0.94 | 0.997 | x | 0,04 |
| *RPA1* | involved in nucleotide excision pathway, binds DNA in preincision complex RPA1 | NM 002945 | 100 | GAAAGACTCACTTGTAGACATCATC | ATTCCTCTTGGCAACTTCTCTG | 0.91 | 0.991 | x | 0,07 |
| *RPA2* | nucleotide excision pathway, binds DNA in preincision complex RPA2 | NM 002946 | 113 | GAAGTGTTCAGAATTGGGAATGTT | GCTGTCATGTCATCTATTTTGTAAAC | 0.95 | 0.996 | x | 0,02 |
| *XPA* | nucleotide excision pathway, binds damaged DNA in preincision complex | NM 000380 | 102 | GGGGTGATATGAAACTCTACTTAAAG | CCTGTCGGACTTCCTTTGC | 0.93 | 0.993 | x | 0,1 |

**Supplementary Figures 1-11.** Electrophoretic gels for RNA integrity from Experion Automated Electrophoresis Station (all samples).

**
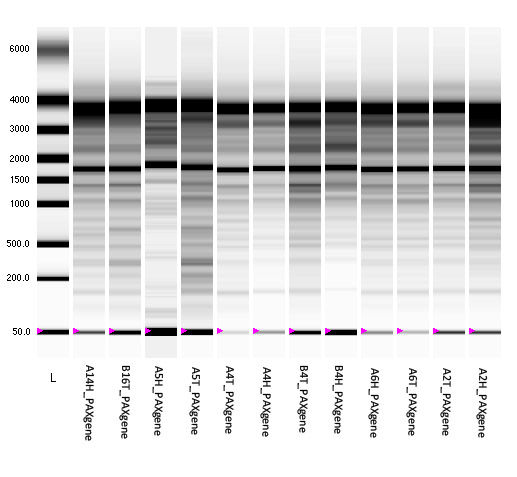
**

**
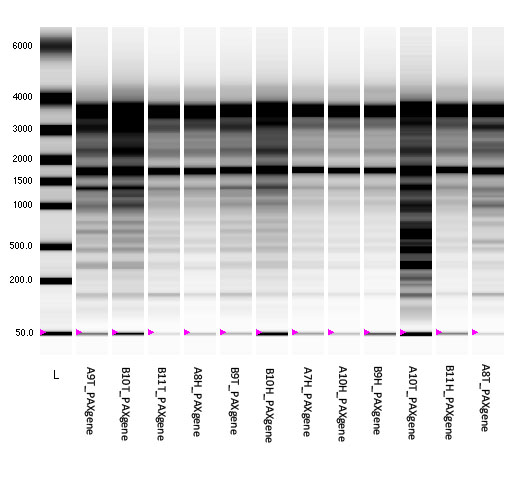
**

**
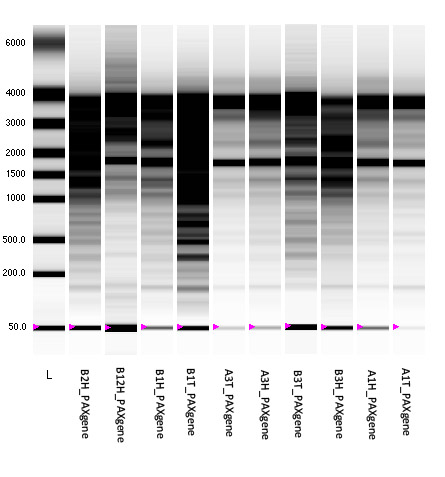
**

**
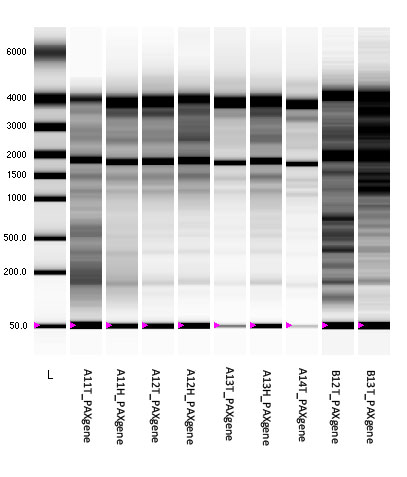
**

**
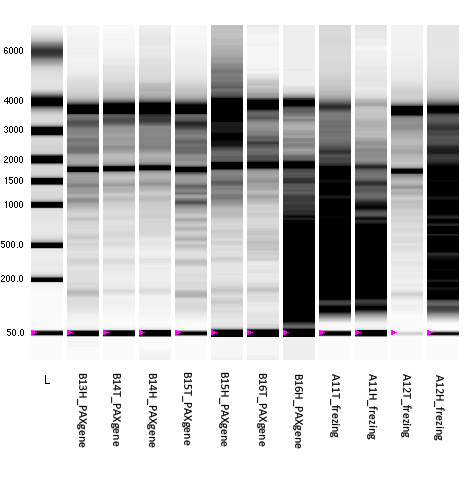
**

**
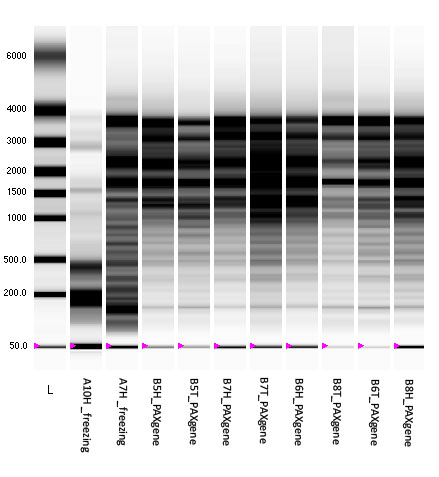
**

**
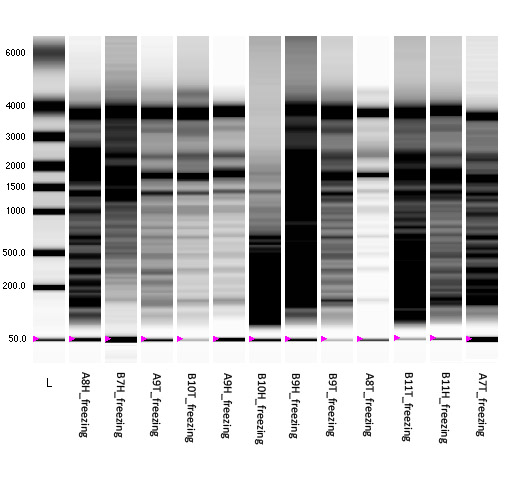
**

**
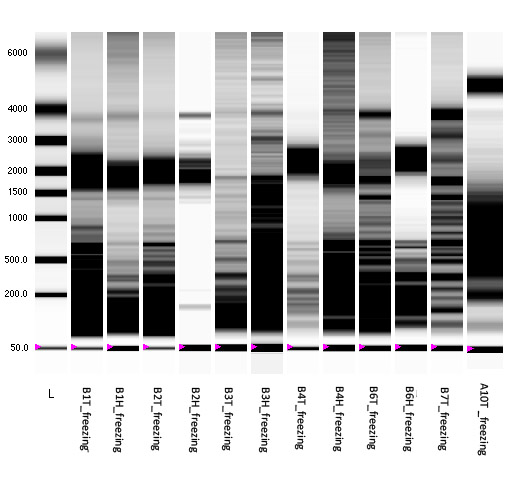
**

**
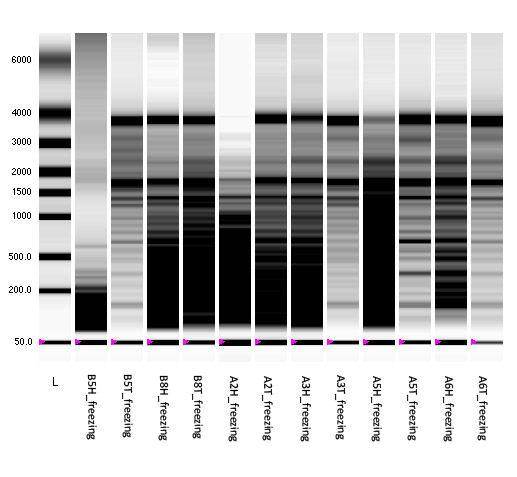
**

**
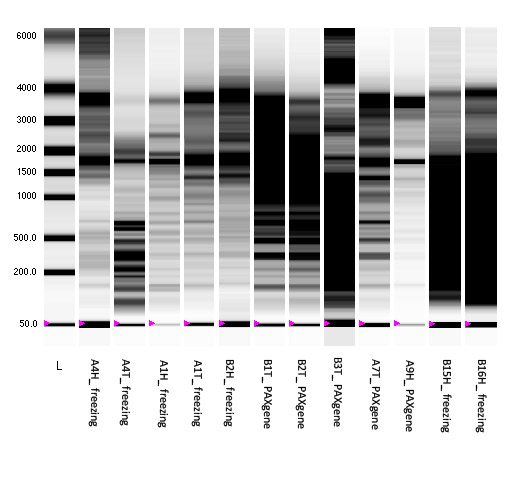
**

**
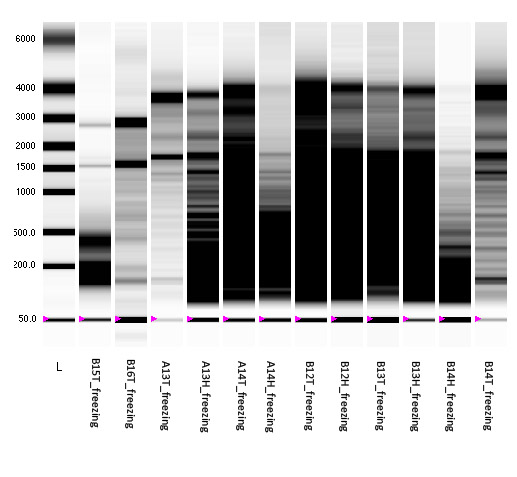
**
